# Supplementary material for: Dynamic Visualization of TGF-β/SMAD3 Transcriptional Responses in Single Living Cells
Source: Cancers (Basel). 2022 May 19;14(10):2508. doi: 10.3390/cancers14102508 (PMC9139966; doi:10.3390/cancers14102508)
Supplement: Supplementary file 1 [file cancers-14-02508-s001.zip › Supplementary Tables S1 and S2.pdf]

Supplementary Table S1: Cloning primers

**Cloning primers**	**5' to 3'**
T7 FW	taatacgactcactataggg
$CAGA_{12}$+MLP FW	tttatcgataggtaccgagctc
CAGA+MLP + age1 REV	cgtataccggtggatcag
D2 domain FW	ggacgagctgtacaagaag
D2 domain REV	ctgcagaattc ttacacattgatcctagcagaagc
eGFP FW	catggtcctgctggagttcgtg
eGFP REV	cgtcgccgtccagctcgaccag
PGK REV	cggtggatgtggaatgtgtg

Supplementary Table S2: rt-qPCR primers

**Gene**	**Forward** (5'→ 3')	**Reverse** (5'→ 3')
GFP	AAGCTGACCCTGAAGTTCATCTGC	TCCAGCAGGACCATGTGATC
dynGFP	GTCGAGCTGGACGGCGACGTA	CACGAACTCCAGCAGGACCATG
Smad7	TGGATGGCGTGTGGGTTTA	TGGCGGACTTGATGAAGATG
Serpine1	GCCAACAAGAGCCAATCACA	AGGCAAGCAAGGGCTGAAG
Ctgf	GGCCTCTTCTGCGATTTCG	CCATCTTTGGCAGTGCACACT
Gapdh	CCAAGTCGGATGTGGAAATGG	TGTCGCAAGTGGACAGTCTC
Hprt	TGGATACAGGCCAGACTTTGTT	CAGATTCAACTTGCGCTCATC
