# Supplementary figures and images for: Dynamic Visualization of TGF-β/SMAD3 Transcriptional Responses in Single Living Cells
Source: Cancers (Basel). 2022 May 19;14(10):2508. doi: 10.3390/cancers14102508 (PMC9139966; doi:10.3390/cancers14102508)

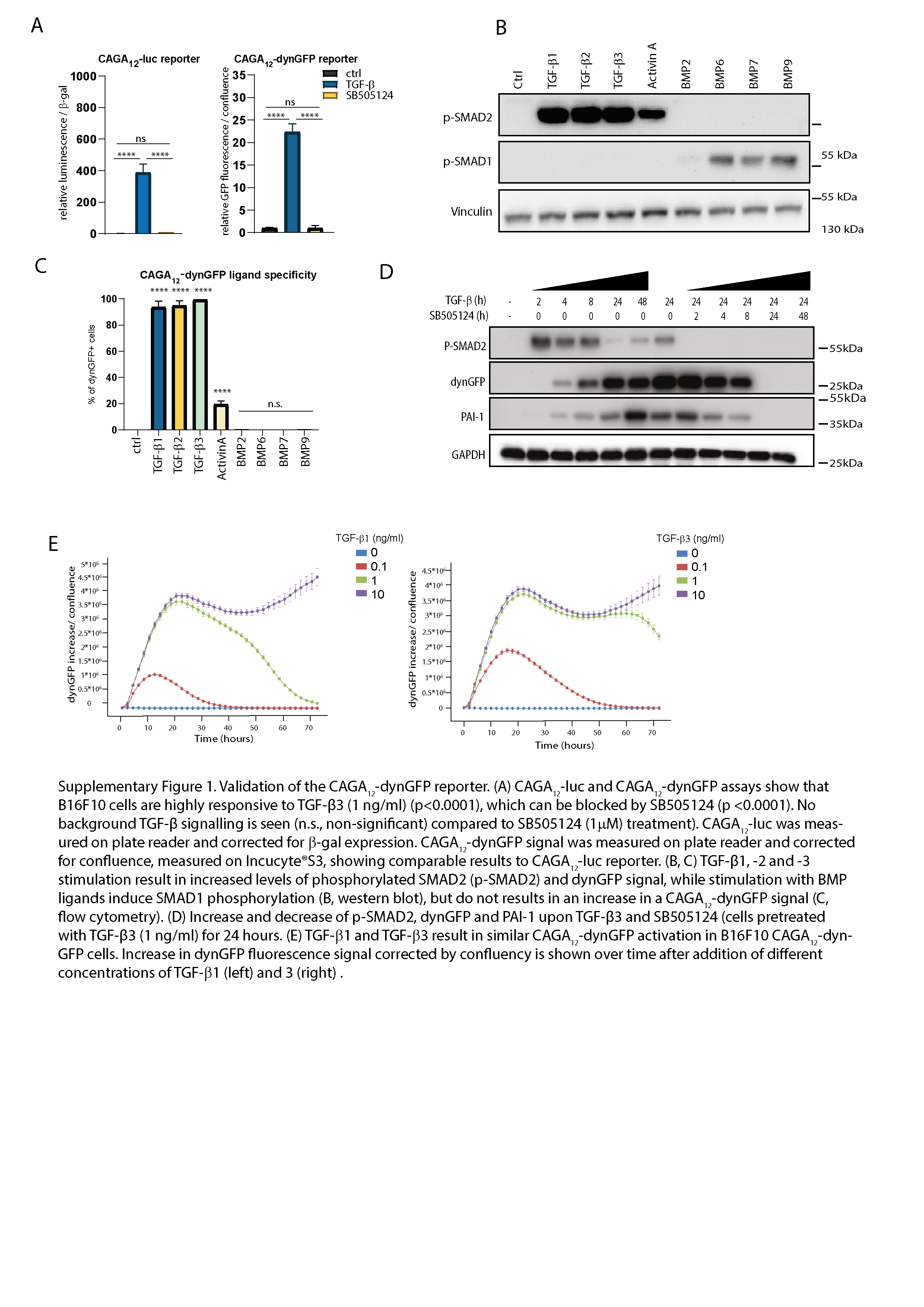

Supplement: Supplementary file 1 [file cancers-14-02508-s001.zip › Figure S1.tif]

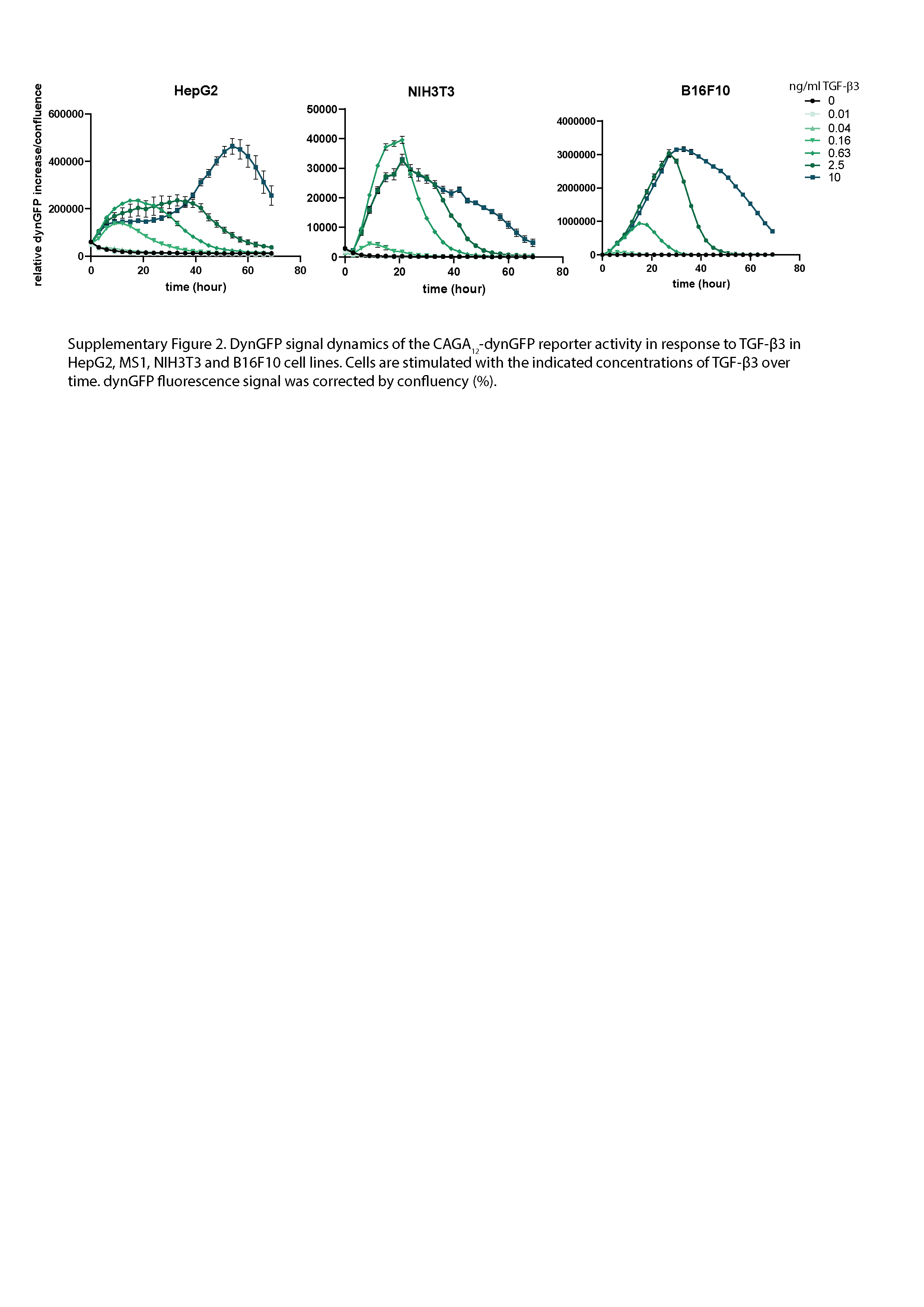

Supplement: Supplementary file 1 [file cancers-14-02508-s001.zip › Figure S2.tif]

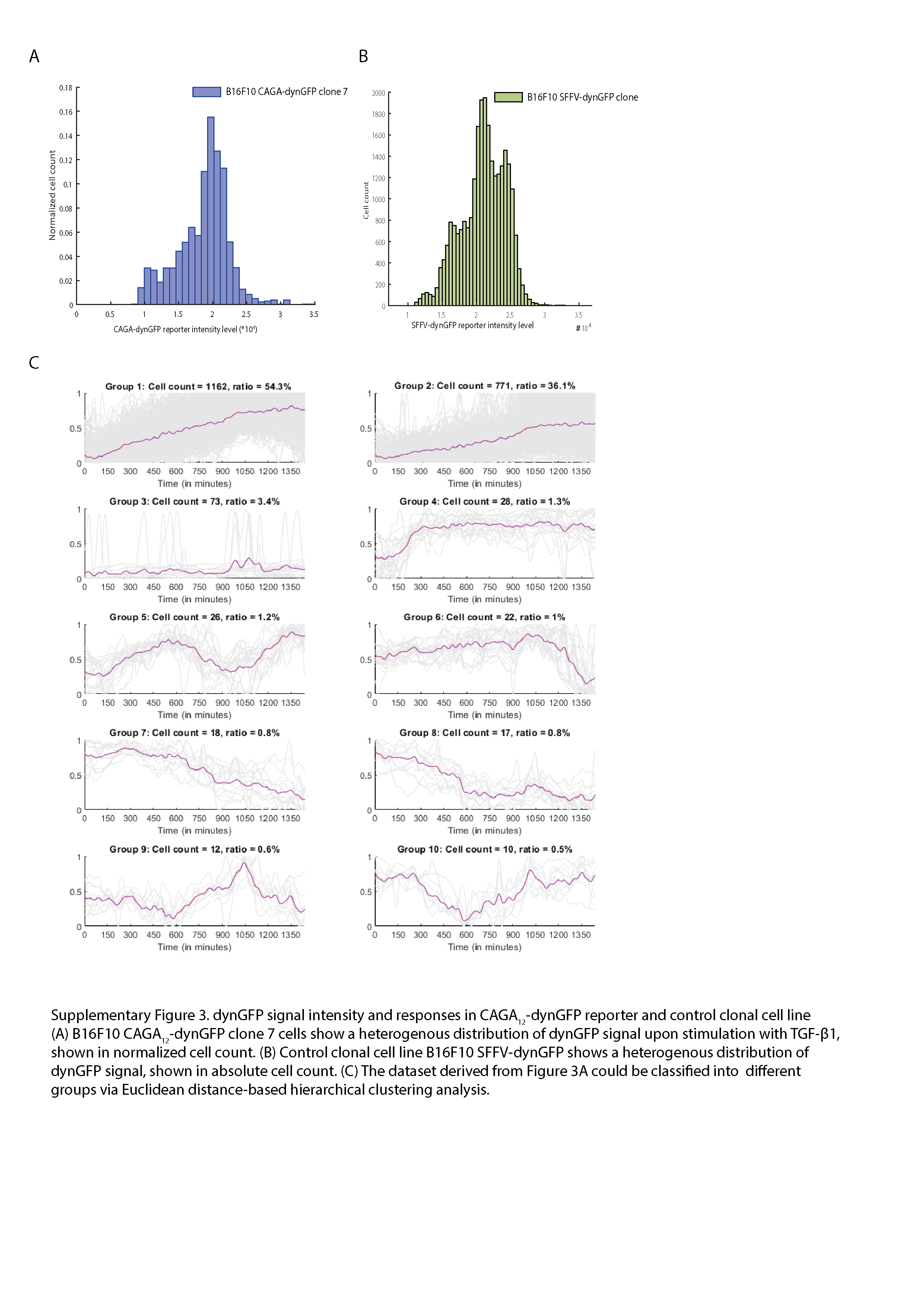

Supplement: Supplementary file 1 [file cancers-14-02508-s001.zip › Figure S3.tif]

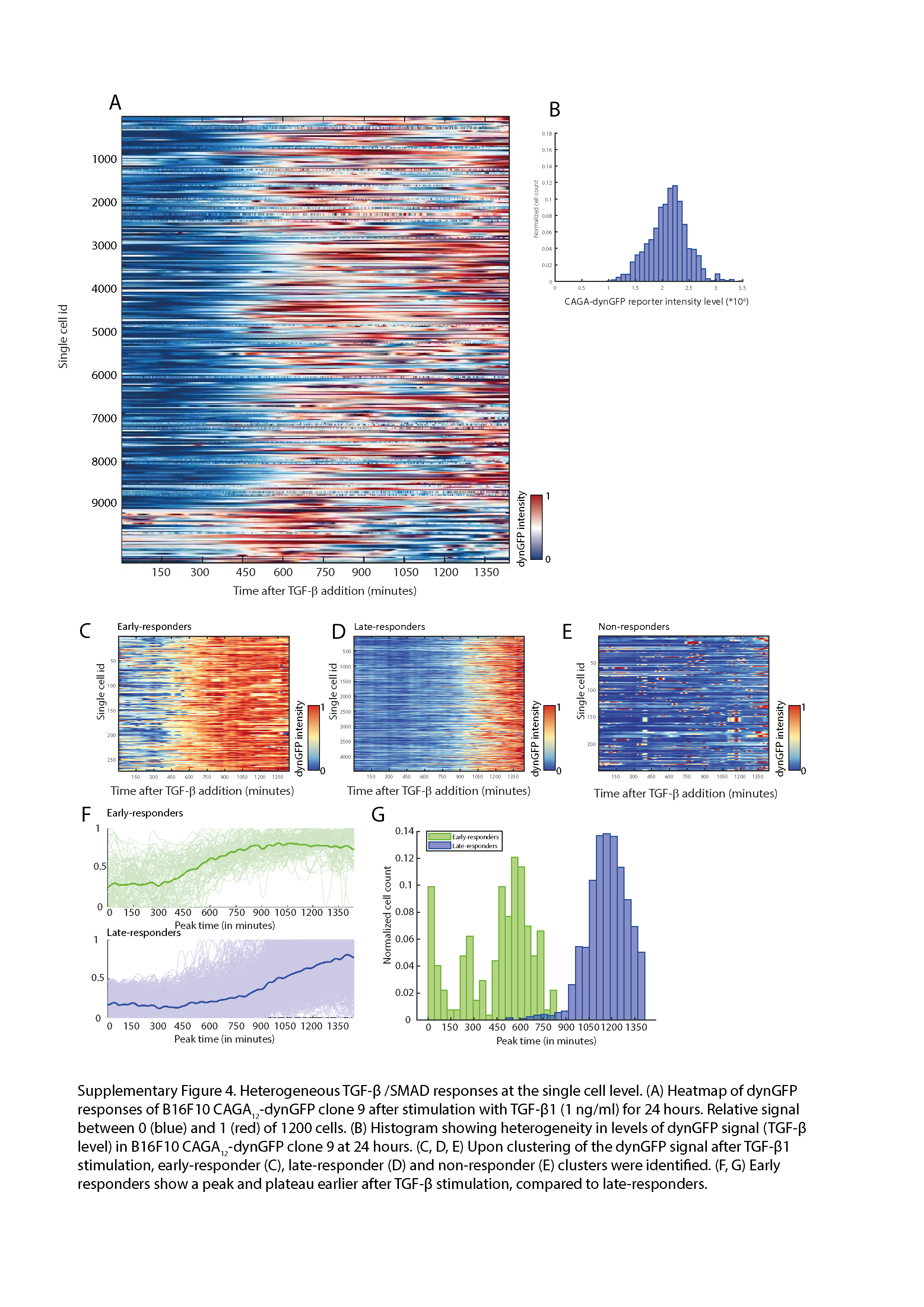

Supplement: Supplementary file 1 [file cancers-14-02508-s001.zip › Figure S4.tif]

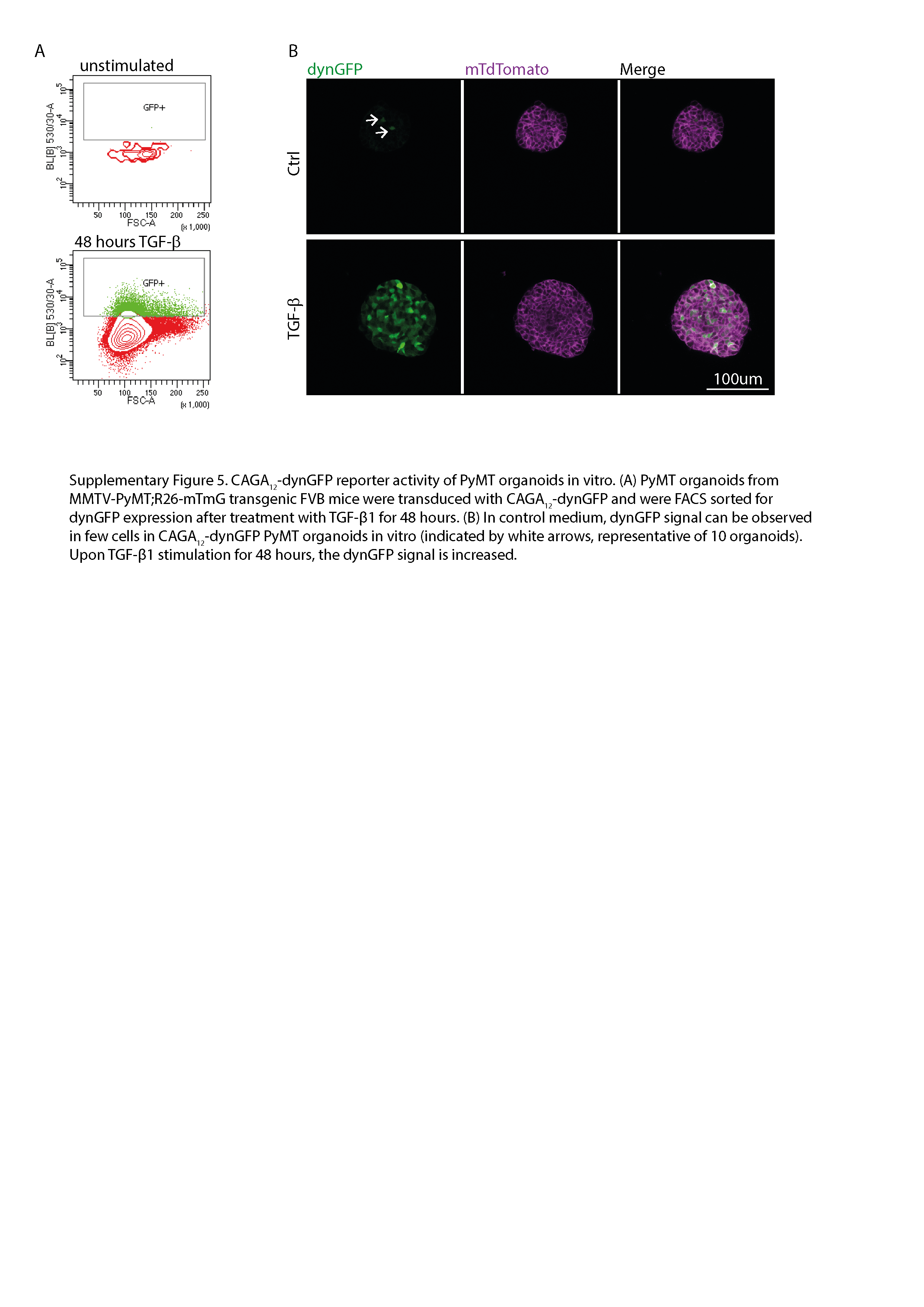

Supplement: Supplementary file 1 [file cancers-14-02508-s001.zip › Figure S5.tif]
